# A meta‐analysis of the association between vasopressor use and intensive care unit‐acquired weakness

**DOI:** 10.1002/brb3.70012

**Published:** 2024-09-05

**Authors:** Tao Yang, Yan Wang, Xiuming Xi, Shanshan Yu

**Affiliations:** ^1^ Department of Critical Care Medicine, Beijing Tiantan Hospital Capital Medical University Beijing China; ^2^ Department of Critical Care Medicine Qingdao Municipal Hospital Qingdao China; ^3^ Department of Critical Care Medicine, Fu Xing Hospital Capital Medical University Beijing China; ^4^ Department of Critical Care Medicine Jinyang Hospital Affiliated of Guizhou Medical University, Guiyang Second People's Hospital Guiyang China

**Keywords:** ICU‐acquired weakness, intensive care unit, meta‐analysis, noradrenaline use, vasopressor use, vasopressors

## Abstract

**Objective:**

This study aims to clarify the uncertain association between vasopressor administration and the development of intensive care unit‐acquired weakness (ICUAW) in critically ill adult patients.

**Methods:**

We conducted a comprehensive search of PubMed, Embase, Web of Science, and the Cochrane Central Register of Controlled Trials up to October 10, 2023. Titles and abstracts were independently screened by two authors, who then reviewed full texts and extracted relevant data from the studies that met the inclusion criteria. This review included prospective and retrospective cohort studies that explored the relationship between vasopressor use and ICUAW utilizing univariate or multivariate analysis in adult ICU patients.

**Results:**

A total of 15 studies were included in our review, collectively indicating a statistically significant association between the use of vasopressors and the occurrence of ICUAW (odds ratio [OR], 3.43; 95% confidence intervals [CI], 1.95–6.04), including studies utilizing multivariate analysis (OR, 3.43; 95% CI, 1.76–6.70). Specifically, the use of noradrenaline was significantly associated with ICUAW (OR, 4.42; 95% CI, 1.69–11.56). Subgroup and sensitivity analyses further underscored the significant relationship between vasopressor use and ICUAW, particularly in studies focusing on patients with clinical weakness, varying study designs, different sample sizes, and relatively low risk of bias. However, this association was not observed in studies limited to patients with abnormal electrophysiology.

**Conclusions:**

Our review underscores a significant link between the use of vasopressors and the development of ICUAW in critically ill adult patients. This finding helps better identify patients at higher risk of ICUAW and suggests considering targeted therapies to mitigate this risk.

## Graphical abstract

This study demonstrates a statistically significant relationship between vasopressor administration and the development of ICU‐acquired weakness in critically ill adult patients.

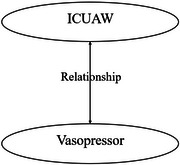

## 1 INTRODUCTION

Intensive care unit‐acquired weakness (ICUAW) represents a prevalent neuromuscular complication in patients experiencing critical illnesses. This condition is notably linked with prolonged mechanical ventilation, escalated healthcare expenditures, extended durations in both intensive care unit (ICU) and hospital settings, and an increase in ICU‐ and hospital‐related mortality rates (Hermans et al., 2014; Nguyen The & Nguyen Huu, 2015; Peñuelas et al., 2018). Septic shock, a significant public health concern, is characterized by elevated mortality and morbidity (Rhodes et al., 2017). In this context, vasopressor therapy emerges as a cornerstone in managing critically ill patients with septic shock (Evans et al., 2021). The incidence of ICUAW is high among this patient population; however, the precise relationship between vasopressor administration and the development of ICUAW remains to be elucidated. The potential adverse effects of vasopressors, particularly concerning ICUAW onset, have garnered considerable attention from researchers and clinicians alike. Although some clinical trials suggest a statistically notable correlation between vasopressor use and ICUAW development, others report no such association. This systematic review and meta‐analysis aim to synthesize data from prospective and retrospective cohort studies to rigorously evaluate the association between vasopressor utilization and the emergence of ICUAW.

## 2 METHODS

This study was performed following the Preferred Reporting Items for Systematic Reviews and Meta‐Analyses: the PRISMA statement (Moher et al., 2009).

### 2.1 Search strategy

The following databases were searched for pertinent English language studies from inception through October 10, 2023: PubMed, Embase, Web of Science, and the Cochrane Central Register of Controlled Trials. We used specific search terms for PubMed (Supporting Information) and adapted them for the other databases. Additionally, we performed a manual search of references cited by the included articles and relevant review articles to identify other eligible studies.

### 2.2 Selection criteria

The inclusion criteria were as follows: prospective and retrospective cohort studies in adult patients (age >18) that evaluated the use of vasopressors and the incidence of ICUAW utilizing univariate or multivariate analysis; diagnoses of ICUAW (Fan et al., 2014; Stevens et al., 2009) made using diagnostic tests (electrophysiological studies, histopathology of muscle, or nerve tissue) or manual muscle testing (Medical Research Council [MRC] weakness scale). Vasopressors refer to noradrenaline, metaraminol, vasopressin, and adrenaline. The exclusion criteria were as follows: patients with primary polyneuropathies (e.g., Guillain‐Barré syndrome, myasthenia gravis) or myopathies (e.g., idiopathic inflammatory myopathies); and studies with insufficient data reported.

### 2.3 Study selection and data abstraction

Two reviewers (T.Y. and Y.W.) independently reviewed studies based on the inclusion criteria. They also independently extracted the following data from each study using a standardized data collection form: author information, publication year, study design, study location, ICUAW incidence, number of participants, tools of neuromuscular evaluation, inclusion and exclusion criteria, number of ICUAW patients given and not given vasopressors, and ICUAW mortality. Authors of the included studies were contacted when data required clarification. Disagreements in study selection or data extraction were resolved by either consensus or a third‐party decision (S.S.Y.).

### 2.4 Study quality assessment

Two reviewers (T.Y. and Y.W.) independently assessed the methodological quality of each study using the Newcastle–Ottawa scale (Higgins & Green, 2015) and the Quality Assessment Tool for Diagnosis Accuracy Studies (QUADAS‐2) (Whiting et al., 2003).

### 2.5 Data analysis

The meta‐analysis was performed using Stata version 15.0 (StataCorp), and the results were analyzed using odds ratios (ORs) and 95% confidence intervals (CIs). The DerSimonian and Laird random effects model was used for data analyses. Heterogeneity was assessed using the *χ* ^2^ statistic with *p* ≤ .1 considered statistically significant. The impact of statistical heterogeneity on the study results was estimated by calculating the *I* ^2^ statistic. Values of the *I* ^2^ statistic above 50% were regarded as a cutoff point for considerable heterogeneity. Subgroup analyses examined (1) studies using clinical muscle testing and electrophysiology as diagnostic methods; (2) studies with relatively large sample sizes (excluding studies with a sample size less than 100); and (3) prospective and retrospective cohort studies. The sensitivity analysis examined studies with a relatively low risk of bias (excluding studies with a Newcastle–Ottawa scale score <7). We examined publication bias using Begg's rank correlation test for quantitative assessment and funnel plots for qualitative assessment.

## 3 RESULTS

### 3.1 Study search and selection

**FIGURE 1:**
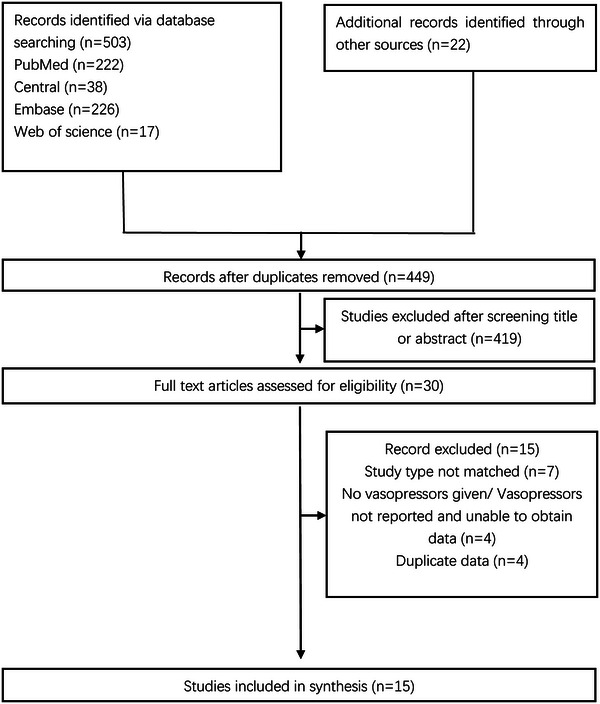
Flow diagram of study selection process.

The initial exploration of the literature resulted in identifying 503 citations, as illustrated in Figure 1. A subsequent thorough examination of these studies’ bibliographies led to the discovery of an additional 22 articles. Following a meticulous screening of titles and abstracts, the selection was narrowed down to 30 articles for a detailed full‐text review. Ultimately, 15 studies (Abdelmalik & Rakocevic, 2017; Brunello et al., 2010; Dres et al., 2019; Frithiof et al., 2021; Hermans et al., 2009; Nanas et al., 2008; Schmidt et al., 2023; Shabana et al., 2021; TEAM Study Investigators et al., 2015; Watanabe et al., 2023; Weber‐Carstens et al., 2010; Wolfe et al., 2018; Yang et al., 2021, 2023; Zhang et al., 2021) met the criteria and were incorporated into this review. Of these, nine studies (Abdelmalik & Rakocevic, 2017; Brunello et al., 2010; Hermans et al., 2009; Nanas et al., 2008; Shabana et al., 2021; Weber‐Carstens et al., 2010; Wolfe et al., 2018; Yang et al., 2021, 2023) used multivariable regression analysis for vasopressors.

### 3.2 Study characteristics and quality

The characteristics of the studies included in this systematic review are detailed in Table 1. Altogether, these studies encompassed 2156 patients. Among them, nine were prospective studies (Brunello et al., 2010; Frithiof et al., 2021; Nanas et al., 2008; Schmidt et al., 2023; TEAM Study Investigators et al., 2015; Weber‐Carstens et al., 2010; Yang et al., 2021, 2023; Zhang et al., 2021), and six were retrospective (Abdelmalik & Rakocevic, 2017; Dres et al., 2019; Hermans et al., 2009; Shabana et al., 2021; Watanabe et al., 2023; Wolfe et al., 2018). The diagnosis of ICUAW in these studies (Frithiof et al., 2021; Hermans et al., 2009; Shabana et al., 2021; Weber‐Carstens et al., 2010) was primarily determined using electrophysiological methods in four studies, whereas the MRC scale was employed in 11 studies (Abdelmalik & Rakocevic, 2017; Brunello et al., 2010; Dres et al., 2019; Nanas et al., 2008; Schmidt et al., 2023; TEAM Study Investigators et al., 2015; Watanabe et al., 2023; Wolfe et al., 2018; Yang et al., 2021, 2023; Zhang et al., 2021). ICU mortality rates varied among the studies.

**TABLE 1:** Characteristics of selected studies.

Study	Study size	Study design	Country	Setting	Population	Examination	ICUAW	Use of VA^a^	ICU mortality (%)^a^
Watanabe (2023)	143	Retrospective study	Japan	MSICU	MV > 48 h	Clinical	62	54 vs. 73	NR
Yang et al. (2023)	280	Prospective study	China	MSICU	ICU‐LOS > 24 h	Clinical	40	19 vs. 69	NR
Schmidt et al. (2023)	68	Prospective study	Brazil	MSICU	ICU‐LOS ≥ 72 h and SARS‐CoV‐2 infection	Clinical	24	21 vs. 28	NR
Frithiof et al. (2021)	111	Prospective study	Sweden	MSICU	SARS‐CoV‐2 infection	EMG	11	11 vs. 40	20% vs. 27%
Zhang et al. (2021)	37	Prospective study	China	MSICU	ICU‐LOS ≥ 2 days	Clinical	37	15 vs. 2	NR
Shabana et al. (2021)	114	Retrospective study	Egypt	MSICU	ICU‐LOS ≥ 7 days	EMG	57	28 vs. 15	NR
Yang et al. (2021)	68	Prospective study	China	MSICU	ICU‐LOS ≥ 7 days	Clinical	30	28 vs. 19	63.3% vs. 21%
Dres et al. (2019)	116	Retrospective study	France	MSICU	MV ≥ 24 h	Clinical	66	51 vs. 12	27.3% vs. 6%
Wolfe et al. (2018)	172	Retrospective study	American	MSICU	MV > 3days	Clinical	80	59 vs. 31	NR
Abdelmalik and Rakocevic (2017)	148	Retrospective study	American	MSICU	ARF and sepsis	Clinical	74	58 vs. 43	NR
TEAM Study Investigators et al. (2015)	94	Prospective study	Australia and New Zealand	MSICU	MV > 3days	Clinical	48	28 vs. 29	NR
Weber‐Carstens et al. (2010)	40	Prospective study	Germany	SICU	MV and SAPS‐ ≥ 20	EMG	22	20 vs. 11	NR
Brunello et al. (2010)	39	Prospective study	Switzerland	MSICU	MV > 48 h and SIRS	Clinical	13	10 vs. 7	77% vs. 33%
Hermans et al. (2009)	541	Retrospective study	Belgium	MSICU	MV > 7 days	EMG	301	232 vs. 197	NR
Nanas et al. (2008)	185	Prospective study	Greece	MSICU	LOS > 10 days	Clinical	44	40 vs. 96	36% vs. 20%

Abbreviations: COPD, chronic obstructive pulmonary disease; EMG, electromyography; ICU, intensive care unit; ICUAW, intensive care unit‐acquired weakness; LOS, length of stay; MICU, medical ICU; MOF, multiple organ failure; MSICU, medical surgical ICU; MV, mechanical ventilation; NR, not reported; SAPS, simplified acute physiology score; SICU, surgical ICU; VA, vasopressors.

^a^ Comparison between ICUAW and no ICUAW.

**FIGURE 2:**
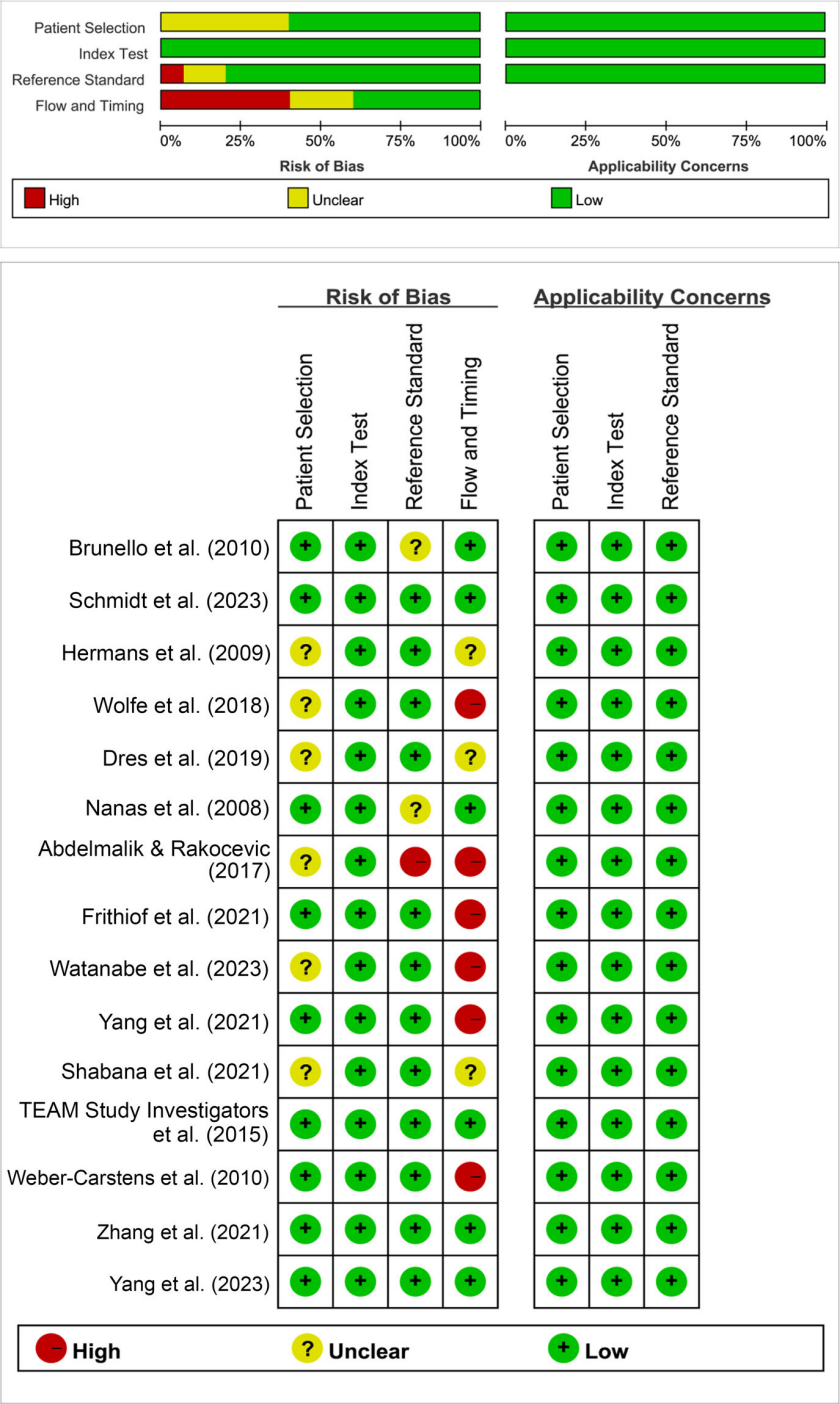
The quality outcomes of the included studies.

The assessment of methodological quality for the included studies is detailed in Table 2. Generally, there was a noticeable risk of bias across the studies. Specifically, not all studies (Dres et al., 2019; Frithiof et al., 2021; Schmidt et al., 2023; TEAM Study Investigators et al., 2015; Watanabe et al., 2023; Zhang et al., 2021) conducted statistical comparisons using multivariable regression analysis for vasopressors, leading to incomplete comparability scores. Additionally, several studies (Abdelmalik & Rakocevic, 2017; Brunello et al., 2010; Nanas et al., 2008) did not clarify whether their assessments were independently blinded for clinicians or physical therapists. Furthermore, there were inconsistencies in cohort follow‐up completeness in some studies (Abdelmalik & Rakocevic, 2017; Frithiof et al., 2021; Hermans et al., 2009; Shabana et al., 2021; Watanabe et al., 2023; Weber‐Carstens et al., 2010; Wolfe et al., 2018; Yang et al., 2021). The quality outcomes of the included studies according to the QUADAS‐2 are shown in Figure 2.

**TABLE 2:** Methodology and reporting assessment.

Newcastle‐Ottawa quality assessment scale for cohort studies									
Studies	Selection				Comparability	Outcome			Score
	Exposed representative	Nonexposed representative	Ascertainment of exposure	Outcome of interest not present at start		Assessment of outcome	Adequate duration of follow‐up	Completeness of follow‐up	
Watanabe et al. (2023)	Y	Y	Y	Y	Y, N	Y	N	N	6
Yang et al. (2023)	Y	Y	Y	Y	N, Y	Y	Y	Y	8
Schmidt et al. (2023)	Y	Y	Y	Y	Y, N	Y	Y	Y	8
Frithiof et al. (2021)	Y	Y	Y	Y	Y, N	Y	N	N	6
Zhang et al. (2021)	Y	Y	Y	Y	N, N	Y	Y	Y	7
Shabana et al. (2021)	Y	Y	Y	Y	Y, Y	Y	Y	N	8
Yang et al. (2021)	Y	Y	Y	Y	Y, Y	Y	N	N	7
Dres et al. (2019)	Y	Y	Y	Y	N, N	Y	Y	Y	7
Wolfe et al. (2018)	Y	Y	Y	Y	N, Y	Y	N	N	6
Abdelmalik and Rakocevic (2017)	Y	Y	Y	Y	Y, Y	N	N	N	6
TEAM Study Investigators et al. (2015)	Y	Y	Y	Y	Y, N	Y	Y	Y	8
Weber‐Carstens et al. (2010)	Y	Y	Y	Y	Y, Y	Y	N	N	7
Brunello et al. (2010)	Y	Y	Y	Y	Y, Y	N	Y	Y	8
Hermans et al. (2009)	Y	Y	Y	Y	Y, Y	Y	N	Y	8
Nanas et al. (2008)	Y	Y	Y	Y	N, Y	N	Y	Y	7

*Note*: Y—criteria satisfied, N—criteria not satisfied.

### 3.3 Vasopressors and ICUAW

**FIGURE 3:**
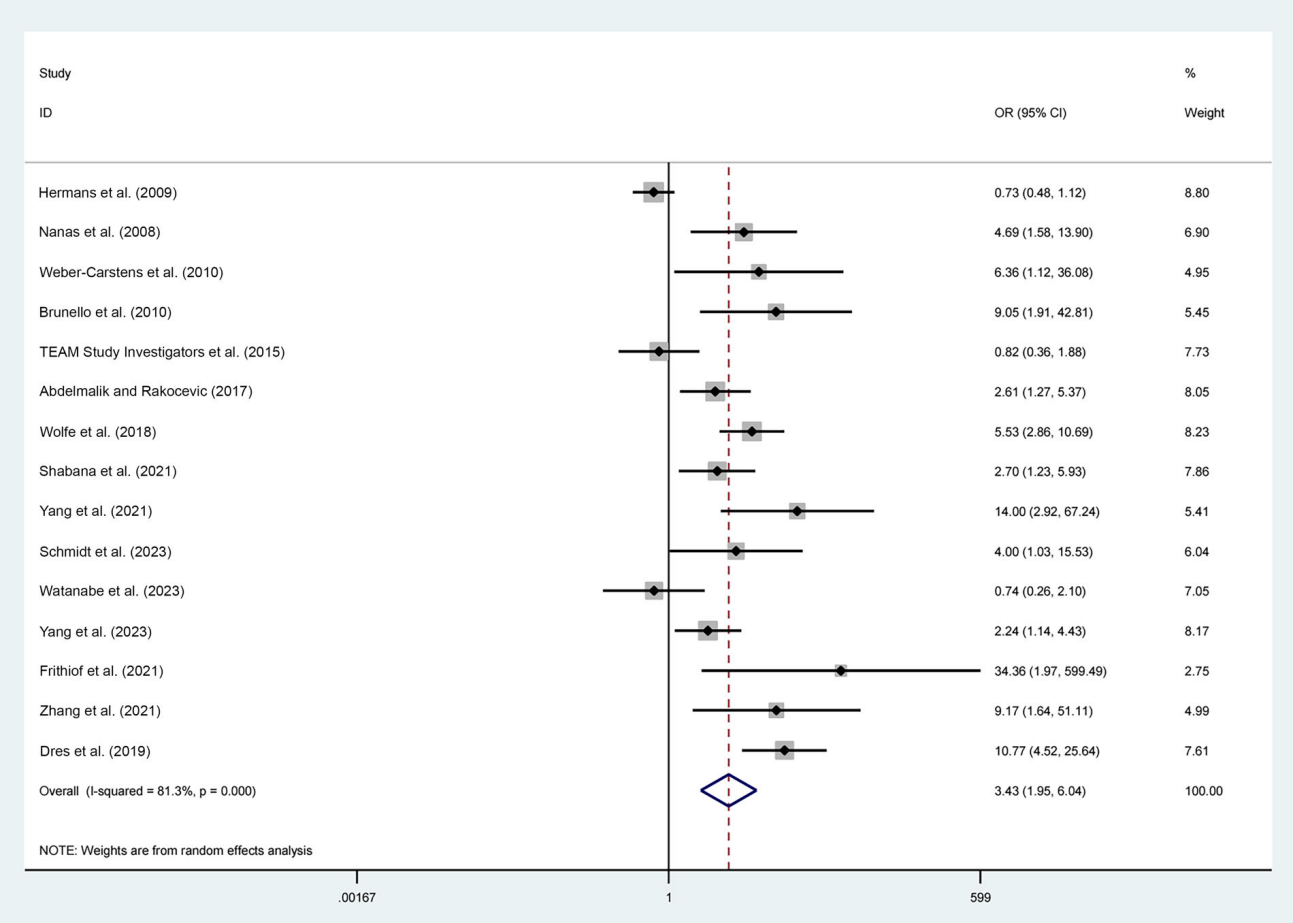
Meta‐analysis of included studies.

When the data from 15 studies were combined, as shown in Figure 3, the analysis found a notable association between the use of vasopressors and the occurrence of ICUAW. The OR was 3.43 with a 95% CI of 1.95–6.04, indicating a statistically significant result (*p* < .01). To address the heterogeneity in the data, a random effects model was applied (*τ* ^2^ = .90; *χ* ^2^ = 74.83, df = 14; *p* < .01; *I* ^2^ = 81.3%). This analysis revealed that the incidence of ICUAW was substantially higher in patients treated with vasopressors (50.1%) compared to those in the control group (27.4%).

Furthermore, when the results from nine studies utilizing multivariate analysis to determine the relationship between vasopressor use and ICUAW were combined, as shown in Table 3, the analysis also found a significant association between the use of vasopressors and the occurrence of ICUAW. The OR was 3.43 with a 95% CI of 1.76–6.70, indicating a statistically significant result (*p* < .01). To address the heterogeneity in the data, a random effects model was applied (*τ* ^2^ = .78; *χ* ^2^ = 45.17, df = 8; *p* < .01; *I* ^2^ = 82.3%). This analysis revealed that the incidence of ICUAW was substantially higher in patients treated with vasopressors (50.3%) compared to those in the control group (27.6%).

**TABLE 3:** Subgroup and sensitivity analyses.

Analyses	*n*	*I* ^2^ (%)	Ph	OR	95% CI	Pe	Incidence vasopressor (%)	Incidence control (%)
Primary analysis	2156 1587 1256	81.3	<.01	3.43	1.95–6.04	<.01	50.1	27.4
Studies utilizing multivariate analysis		82.3	<.01	3.43	1.76–6.70	<.01	50.3	27.6
Studies with use of noradrenaline		88.8	<.01	4.42	1.69–11.56	<.01	54.8	28.2
Diagnostic method								
Studies using clinical assessment	1350 806	72.7	<.01	3.64	2.06–6.43	<.01	48.4	21.9
Studies using electrophysiology		83.3	<.01	2.89	.80–10.44	.11	52.5	39.7
Study type								
Prospective studies	922 1234	63.1	<.01	4.45	2.19–9.04	<.01	38.9	14.9
Retrospective studies		89.9	<.01	2.46	1.00–6.07	.05	56.5	41.5
Sample size								
Studies with *n* ≥ 100	1810 346 1582	85.6	<.01	2.91	1.44–5.86	<.01	48.9	26.8
Studies with *n* < 100		71.5	<.01	4.83	1.65–14.09	<.01	56.0	30.4
Sensitivity analysis (studies with NOS ≥7)		82.9	<.01	3.61	1.80–7.25	<.01	50.4	29.3

Abbreviations: CI, confidence intervals; *I* ^2^, *I*‐squared statistic test for heterogeneity; NOS, the Newcastle–Ottawa scale score; OR, odds ratio; Pe, *p* value for the effect estimate for each subgroup; Ph, *p* value for test of heterogeneity.

### 3.4 Noradrenaline and ICUAW

Upon analyzing the outcomes of seven studies (Brunello et al., 2010; Dres et al., 2019; Hermans et al., 2009; Weber‐Carstens et al., 2010; Wolfe et al., 2018; Yang et al., 2021, 2023) that utilized noradrenaline, a significant link was observed between its use and an increased likelihood of ICUAW development. The effect size, with an OR of 4.42 and a 95% CI ranging from 1.69 to 11.56 (*p* < .01), highlights this significant association. This pooled analysis, employing a random effects model due to notable heterogeneity (*τ* ^2^ = 1.37; *χ* ^2^ = 53.72, df = 6; *p* < .01; *I* ^2^ = 88.8%), revealed an ICUAW incidence of 54.8% in the noradrenaline group, contrasted with 28.2% in the control group.

### 3.5 Subgroup analyses

#### 3.5.1 Clinical assessment versus electrophysiology

The subgroup analyses, detailed in Table 3, include results from eleven studies (Abdelmalik & Rakocevic, 2017; Brunello et al., 2010; Dres et al., 2019; Nanas et al., 2008; Schmidt et al., 2023; TEAM Study Investigators et al., 2015; Watanabe et al., 2023; Wolfe et al., 2018; Yang et al., 2021, 2023; Zhang et al., 2021) that investigated vasopressor use in relation to clinical weakness. These studies found a higher event rate of 48.4% in the vasopressor group compared to 21.9% in the control group. The pooled effect size (OR 3.64; 95% CI 2.06–6.43; *p *< .01) revealed a significant association with a random effects model considering the heterogeneity (*τ* ^2^ = .63; *χ* ^2^ = 36.57, df = 10 (*p* < .01); *I* ^2^ = 72.7%). This analysis demonstrated a substantial connection between vasopressor use and increased clinical weakness. However, a separate analysis of four studies (Frithiof et al., 2021; Hermans et al., 2009; Shabana et al., 2021; Weber‐Carstens et al., 2010) focusing on abnormal electrophysiology showed contrasting results, with an incidence rate of 52.5% in the vasopressor group versus 39.7% in the control group, but without a statistically significant association (OR 2.89; 95% CI.80–10.44; *p* = .11). Data were pooled using a random effects model considering the observed heterogeneity (*τ* ^2^ = 1.21; *χ* ^2^ = 17.98, df = 3 (*p* < .01); *I* ^2^ = 83.3%).

#### 3.5.2 Sample sizes (*n* ≥ 100 vs. *n* < 100)

Upon analyzing the outcomes of nine studies (Abdelmalik & Rakocevic, 2017; Dres et al., 2019; Frithiof et al., 2021; Hermans et al., 2009; Nanas et al., 2008; Shabana et al., 2021; Watanabe et al., 2023; Wolfe et al., 2018; Yang et al., 2023) with sample sizes exceeding 100, a significant link was established between vasopressor use and ICUAW, as indicated by an OR of 2.91 (95% CI: 1.44–5.86; *p* < .01). This analysis, utilizing a random effects model due to notable heterogeneity (*τ* ^2^ = .90; *χ* ^2^ = 55.74, df = 8, *p *< .01, *I* ^2^ = 85.6%), showed an ICUAW incidence of 48.9% in the vasopressor group compared to 26.8% in the control group. In contrast, the six studies (Brunello et al., 2010; Schmidt et al., 2023; TEAM Study Investigators et al., 2015; Weber‐Carstens et al., 2010; Yang et al., 2021; Zhang et al., 2021) with smaller sample sizes (less than 100) displayed a higher unadjusted incidence rate of 56.0% in the vasopressor group versus 30.4% in the control group, reinforcing the association with an OR of 4.83 (95% CI: 1.65–14.09; *p* < .01) with a random effects model considering the observed heterogeneity (*τ* ^2^ = 1.24; *χ* ^2^ = 17.54, df = 5 (*p* < .01); *I^2^* = 71.5%).

#### 3.5.3 Prospective versus retrospective cohort studies

This meta‐analysis, incorporating data from nine prospective studies (Brunello et al., 2010; Frithiof et al., 2021; Nanas et al., 2008; Schmidt et al., 2023; TEAM Study Investigators et al., 2015; Weber‐Carstens et al., 2010; Yang et al., 2021, 2023; Zhang et al., 2021), revealed a significant association between the use of vasopressors and the incidence of ICUAW. Utilizing a random effects model to account for observed heterogeneity (*τ* ^2^ = .67; *χ* ^2^ = 21.69, df = 8 (*p* < .01) *I* ^2^*^^* = 63.1%), the analysis reported an OR of 4.45 (95% CI, 2.19–9.04; *p* < .01). The incidence of ICUAW was notably higher in patients receiving vasopressors, at 38.9%, compared to 14.5% in the control group. Further analysis, which included results from six retrospective studies (Abdelmalik & Rakocevic, 2017; Dres et al., 2019; Hermans et al., 2009; Shabana et al., 2021; Watanabe et al., 2023; Wolfe et al., 2018), continued to underscore a significant link between vasopressor administration and ICUAW. With an OR of 2.46 (95% CI, 1.00–6.07; *p* = .05), this pooled effect size was calculated using a random effects model, again considering the observed heterogeneity (*τ* ^2^ = 1.12; *χ* ^2^ = 49.30, df = 5 (*p *< .01) *I* ^2^ = 89.9%). The comparative incidence of ICUAW escalated to 56.5% in the vasopressor group, against 41.5% in the control group.

### 3.6 Sensitivity analysis

The sensitivity analysis, as detailed in Table 3, maintains the significant association between vasopressor administration and the incidence of ICUAW, even after the exclusion of studies identified with a high risk of bias. The remaining studies (Brunello et al., 2010; Dres et al., 2019; Hermans et al., 2009; Nanas et al., 2008; Schmidt et al., 2023; Shabana et al., 2021; TEAM Study Investigators et al., 2015; Weber‐Carstens et al., 2010; Yang et al., 2021, 2023; Zhang et al., 2021) reported an OR of 3.61 (95% CI 1.80–7.25; *p* < .01). This analysis employed a random effects model to account for observed heterogeneity (*τ* ^2^ = 1.04; *χ* ^2^ = 58.46, df = 10 (*p* < .01); *I* ^2 ^= 82.9%). The incidence of ICUAW was significantly higher in the vasopressor group at 50.4% compared to 29.3% in the control group not exposed to vasopressors.

### 3.7 Heterogeneity

The analysis identified methodological heterogeneity across the included studies, attributed to variations in diagnostic methods, study design types, and sample sizes. The studies employed either clinical assessment or electrophysiology for diagnoses and were categorized as either prospective or retrospective cohort studies. Furthermore, studies were classified based on sample size, with a cutoff of 100 subjects distinguishing small from large studies. This methodological diversity necessitated three distinct comparisons within the review: clinical assessment versus electrophysiology, prospective versus retrospective studies, and analysis based on sample size (*n* ≥ 100 vs. *n* < 100). Given the high levels of statistical heterogeneity observed within each comparison, a random‐effects model was deemed more appropriate than a fixed‐effects model to adequately address the heterogeneity.

### 3.8 Assessment of publication biases

**FIGURE 4:**
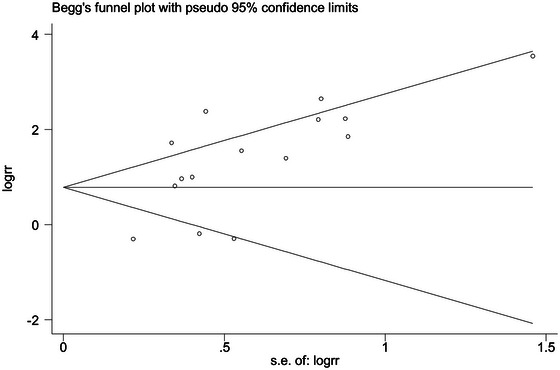
Funnel plots of included studies.

Publication bias was evaluated using funnel plots, as illustrated in Figure 4, which did not exhibit significant asymmetry, suggesting an absence of publication bias. Begg's test further supported this finding (*Z* = 1.78; *p* = .08), indicating no significant publication biases in the meta‐analysis.

## 4 DISCUSSION

This comprehensive review sheds light on the intricate relationship between the use of vasopressors and the development of ICUAW, underlining the significance of vasopressor administration as a notable risk factor for ICUAW. Amid the backdrop of sepsis and septic shock being predominant contributors to healthcare costs and mortality in hospital settings, the role of hemodynamic stabilization emerges as a critical component in managing patients with sepsis or septic shock. This involves initial fluid administration followed by the administration of vasoactive agents in cases of persistent circulatory shock. Despite adequate fluid resuscitation, the necessity for vasopressors to either restore or maintain an optimal mean arterial pressure (MAP) is evident (Vincent, 2022), with current guidelines (Evans et al., 2021) advocating for a target MAP of at least 65 mmHg in patients unresponsive to initial fluid therapy. The variable impact of vasopressors on regional, systemic, and organ perfusion is acknowledged, positing a potential pathophysiological pathway to ICUAW through the diminution of microcirculation (Boerma & Ince, 2010; Di Giantomasso et al., 2003, 2005). This reduction in microcirculation may precipitate bioenergetic failure, culminating in ischemic muscle fiber injury (Boërio et al., 2018; Friedrich et al., 2015). Norepinephrine, recognized as the first‐line vasopressor for shock management, is associated with lower mortality rates and fewer adverse effects (Colling et al., 2018; Evans et al., 2021), yet its correlation with an increased risk of developing ICUAW underscores a critical area of concern. This association is further supported by animal studies suggesting that the elevation of MAP with norepinephrine during resuscitation may exacerbate sepsis‐induced muscle damage, likely through impaired microcirculation and consequent ischemic risk to muscle tissues (Boërio et al., 2018). Vasopressors are used as a potentially life‐saving treatment in patients at high short‐term risk of death from shock. Although it is vitally important to study the long‐term effects of the treatments provided in the ICU, the reason is to help us better identify patients at risk of developing these effects. In the case of ICUAW, clarifying that vasopressor use is a true risk factor for ICUAW may help us better identify patients at higher risk and consider providing targeted therapies aimed at mitigating this risk. There have been studies (Anekwe et al., 2020; Rosa et al., 2023; Wang et al., 2022) suggesting that early activity, rehabilitation training, and functional recovery exercise in severe patients can promote the synthesis of muscle protein, reduce the catabolism of muscle protein, and enhance muscle strength, which is effective in preventing the occurrence and development of ICUAW, and the earlier the intervention, the better the effect. There have also been studies (Liu et al., 2020; Zhou et al., 2022) demonstrating that early enteral nutrition can inhibit the excessive immune response and reduce the incidence of ICUAW.

Subgroup analyses illuminate a significant correlation between vasopressor usage and muscle weakness, particularly in patients presenting with clinical weakness as opposed to those identified through abnormal electrophysiology. This distinction underscores the utility of clinical examinations in diagnosing ICUAW, despite potential limitations in early disease stages due to suboptimal patient consciousness or attentiveness. The sensitivity of electrophysiological studies in detecting subclinical ICUAW introduces an alternative explanation for the disparate outcomes observed between subgroups. Our subgroup analyses revealed that studies limited to different sample sizes, prospective or retrospective cohort, and relatively low risk of bias still demonstrated a significant association between vasopressor use and ICUAW. This result partly demonstrates the stability of the overall effect size.

The review's robustness is evident in subgroup analyses across various dimensions, including sample sizes and study designs, affirming a consistent association between vasopressor use and ICUAW. However, the review is not without limitations. No randomized controlled trials met the inclusion criteria, which diminishes the strength of evidence and recommendations. High levels of heterogeneity were identified for all of the outcomes. We analyzed the outcomes in subgroups classified by study design, diagnostic methods, and sample sizes in an effort to reduce methodological and clinical heterogeneity; however, substantial statistical heterogeneity remained despite these attempts.

## 5 CONCLUSION

This review conclusively demonstrates a statistically significant association between the use of vasopressors, particularly noradrenaline, and the incidence of ICUAW. It underscores the importance for ICU medical teams to coordinate multidisciplinary care to optimize rehabilitation delivery, nutritional support, and early mobilization protocols in patients undergoing vasopressor treatment. Future research should focus on elucidating the mechanisms underlying skeletal muscle dysfunction in patients using vasopressors and developing interventions to mitigate ICUAW.

## AUTHOR CONTRIBUTIONS

**Tao Yang**: Conceptualization; methodology; software; data curation; formal analysis; validation; investigation; writing—original draft. **Yan Wang**: Conceptualization; methodology; software; data curation; formal analysis; validation; investigation; writing—original draft. **Xiuming Xi**: Supervision; investigation; visualization; formal analysis. **Shanshan Yu**: Funding acquisition; supervision; visualization; project administration; resources; writing—review and editing.

## CONFLICT OF INTEREST STATEMENT

The authors declare no conflicts of interest.

### PEER REVIEW

The peer review history for this article is available at https://publons.com/publon/10.1002/brb3.70012.

## Supporting information

Supporting Information

## Data Availability

All data generated or analyzed during this study are included in this published article.
